# Gold Nanoparticle-Enhanced Recombinase Polymerase Amplification for Rapid Visual Detection of *Mycobacterium tuberculosis*

**DOI:** 10.3390/bios15090607

**Published:** 2025-09-15

**Authors:** Sukanya Saikaew, Sirikwan Sangboonruang, Rodjana Pongsararuk, Prapaporn Srilohasin, Bordin Butr-Indr, Sorasak Intorasoot, Ponrut Phunpae, Chayada Sitthidet Tharinjaroen, Surachet Arunothong, Wutthichai Panyasit, Angkana Chaiprasert, Khajornsak Tragoolpua, Usanee Wattananandkul

**Affiliations:** 1 Office of Research Administration, Chiang Mai University, Chiang Mai 50200, Thailand; sukanya.saikaew@cmu.ac.th (S.S.); sirikwan.sang@cmu.ac.th (S.S.); 2 Division of Clinical Microbiology, Department of Medical Technology, Faculty of Associated Medical Sciences, Chiang Mai University, Chiang Mai 50200, Thailand; bordin.b@cmu.ac.th (B.B.-I.); sorasak.in@cmu.ac.th (S.I.); ponrut.p@cmu.ac.th (P.P.); chayada.si@cmu.ac.th (C.S.T.); khajornsak.tr@cmu.ac.th (K.T.); 3 Office of Disease Prevention and Control Region 1 (ODPC-1) Chiang Mai, Department of Disease Control, The Ministry of Public Health, Muang District, Chiang Mai 50100, Thailand; rojjj.suya@gmail.com (R.P.); lek_surachet@yahoo.com (S.A.); dcc.labodpc1@gmail.com (W.P.); 4 Department of Microbiology, Faculty of Medicine Siriraj Hospital, Mahidol University, Bangkok 10700, Thailand; prapaporn.srl@mahidol.ac.th; 5 Drug Resistant Tuberculosis Research (DR-TB) Fund, Siriraj Foundation, Bangkok 10700, Thailand; angkana.cha@mahidol.ac.th; 6 Infectious Diseases Research Unit (IDRU), Faculty of Associated Medical Sciences, Chiang Mai University, Chiang Mai 50200, Thailand; 7 Office for Research and Development, Faculty of Medicine Siriraj Hospital, Mahidol University, Bangkok 10700, Thailand

**Keywords:** Tuberculosis, gold nanoparticle (AuNP), point-of-care diagnosis, resource-limited settings, global health challenge

## Abstract

Tuberculosis (TB) remains a major global health challenge, particularly in resource-limited settings where access to rapid and reliable diagnostics is limited. Conventional diagnostic methods, such as smear microscopy and culture, are either time-consuming or lack adequate sensitivity. This study optimized recombinase polymerase amplification (RPA) using 16 primer combinations targeting IS*6110* highly specific to the *Mycobacterium tuberculosis* complex (MTC). A novel naked-eye assay, TB-GoldDx, was developed by integrating RPA combined with gold nanoparticles (AuNPs), enabling equipment-free diagnostics. TB-GoldDx demonstrated a detection limit of 0.001 ng of MTB H37Rv DNA (~210 bacilli) per 25 µL reaction. Among 100 bacterial strains, it achieved 95.83% sensitivity and 100% specificity among 100 bacterial strains, comprising 72 MTB isolates and 28 nontuberculous bacterial species. In 140 sputum samples, the assay showed 81.43% sensitivity and 58.57% specificity versus acid-fast bacilli (AFB) smear microscopy, with sensitivity improving to 95.45% in high-load AFB 3+ specimens. Compared to a commercial line probe assay (LPA), TB-GoldDx exhibited slightly higher sensitivity (84.78% vs. 82.61%) but lower specificity (54.05% vs. 78.38%). Delivering rapid, visual results in under an hour, TB-GoldDx offers a low-cost, easily deployable solution for point-of-care tuberculosis detection, especially in underserved regions, reinforcing global End TB efforts.

## 1. Introduction

Tuberculosis (TB) continues to pose a global health challenge, especially in high-burden countries. The WHO Global Tuberculosis Report 2024 estimates that 10.8 million individuals developed TB in 2023. Despite Thailand’s ongoing control efforts, it remains one of the 30 high-burden countries for TB and TB/HIV. In 2023, approximately 113,000 TB cases and 11,000 deaths were reported, with only 1,100 laboratory-confirmed cases—yielding a detection rate of 37.9% [1]. This significant diagnostic gap highlights the critical need for accessible, rapid, and reliable molecular tools to improve case detection and reduce transmission in resource-limited settings. Traditional TB diagnostics include acid-fast bacilli (AFB) smear microscopy, which has low sensitivity (detection limit of ~5,000–10,000 bacilli/mL) and cannot distinguish *Mycobacterium tuberculosis* (MTB) from nontuberculous mycobacteria (NTM) [2]. Culture remains the gold standard due to its high sensitivity (detecting as few as 10–100 bacilli/mL) and its ability to differentiate MTB from NTM [3] though it is time-consuming (requiring 2–8 weeks), prone to contamination, and requires biosafety level 3 (BSL-3) laboratory conditions [4,5] Additionally, culture techniques are labor-intensive, susceptible to contamination, and often not feasible in peripheral or resource-limited settings. Delays in obtaining culture results can hinder timely diagnosis and treatment initiation, increasing the risk of disease transmission and poor patient outcomes.

Several molecular diagnostic methods offer faster alternatives to conventional techniques for MTB detection. These include Xpert MTB/RIF (~2 h), line probe assays (LPAs; ~24–48 h), TrueNat MTB (~1 h), and COBAS TaqMan MTB (~2.5 h) [6,7,8]. Despite their improved turnaround times, these methods require reliable electricity, expensive equipment and reagents, and routine maintenance, which limits their deployment in resource-limited settings.

Recently, isothermal nucleic acid amplification technologies have emerged as promising alternatives to conventional molecular methods for point-of-care (POC) tuberculosis diagnostics. Among them, recombinase polymerase amplification (RPA) has been particularly attractive for POC use due to its rapid amplification time (10–30 min) and low temperature operation (37–42 °C). Its procedural simplicity and versatility across various detection platforms, such as colorimetric assay, fluorescence-based readout, lateral flow strips, and real-time monitoring systems [9]. These features enable cost-effective, rapid, and field-adaptable diagnostics, making RPA highly suitable for deployment in low-resource and field-based diagnostic settings [10].

Building on these advantages, several studies have explored the application of RPA for rapid MTB or MTBC detection and identification from clinical isolates and specimens. RPA assays have been developed to target several *Mycobacterium tuberculosis* (MTBC)-specific genes and genomic elements, including IS*6110*, IS*1081*, *Rv3618*, *mts90*, and *Ag85B* [10,11,12,13,14]. Notably, assays targeting IS*6110*-targeted RPA assays have been adapted to diverse detection formats, such as fluorescence-based monitoring, SYBR Green I visual readout, and lateral flow (LF) strip detection [10,11,12]. For example, Singpanomchai et al. reported high sensitivity and specificity for an RPA incorporating SYBR Green I for visual detection of MTB using genomic DNA strains, although the assay requires a relatively high limit of detection (LOD) for IS*6110* [11]. More recently, Sciaudone et al. demonstrated that RPA can be implemented as either real-time RPA (RT-RPA) and lateral flow RPA (LF-RPA) assays targeting IS*6110* to directly detect MTBC in sputum specimens. While RT-RPA achieved high sensitivity and specificity, LF-RPA exhibited lower sensitivity but maintained comparable specificity [12]. Collectively, these findings highlight RPA’s potential as a robust, rapid, and versatile diagnostic tool for TB detection in both clinical and field settings.

Colorimetric methods have been increasingly applied to enhance rapid visual readouts of RPA end-point products detectable by the unaided eye. SYBR Green I, for example, fluoresces upon binding to double-stranded DNA and has been used to visualize MTB-specific RPA products [9]. Gold nanoparticle (AuNP)-based visual assays present an effective alternative for point-of-care (POC) tuberculosis diagnosis. Target-induced aggregation alters surface plasmon resonance, resulting in a visible color change from red to blue [15,16]. Kooti et al. developed a PCR-based tuberculosis assay targeting the IS*6110* sequence, combined with a modified AuNP colorimetric probe-based biosensor. This initiating design enables naked-eye detection of TB through a visible color change, offering a rapid and accessible diagnostic approach [17]. However, many types of RPA-based TB diagnostics rely on fluorescence readers, lateral flow cassettes, or microfluidic cartridges that increase cost, require consumables, and limit use outside centralized laboratories. To address these limitations, we developed TB-GoldDx, an RPA-based assay enhanced with unmodified AuNPs for visual detection. The assay was evaluated on a comprehensive bacterial panel and routine sputum samples from northern Thailand, demonstrating its potential as a practical and accessible diagnostic tool for TB in resource-limited settings.

## 2. Materials and Methods

### 2.1. MTB and Nontuberculous Bacterial Isolates Used in This Study

A total of 100 bacterial strains were evaluated in this study, comprising 72 *M. tuberculosis* (MTB) clinical isolates, 13 nontuberculous mycobacteria (NTM), and 15 non-mycobacterial clinical strains (Table 1). MTB isolates were collected from two sources: 20 from the Office of Disease Prevention and Control 1 (ODPC-1) Chiang Mai, Chiang Mai, Thailand, during the year 2023–2024 and 52 from the Drug-Resistant Tuberculosis Research (DR-TB) Fund, Siriraj Foundation, Bangkok, Thailand. The reference strain *M. tuberculosis* H37Rv (ATCC 27294) was included for assay optimization and validation. Additionally, NTM and non-mycobacterial bacterial strains, including both clinical isolates and standard reference strains from the ODPC-1 Chiang Mai and the Division of Clinical Microbiology (CMB), Faculty of Associated Medical Sciences, Chiang Mai University, Chiang Mai, Thailand, were employed to assess the specificity and cross-reactivity of the assay.

### 2.2. Sputum Specimens

In this study, 140 sputum specimens from individuals suspected of having pulmonary TB were obtained from ODPC-1 Chiang Mai, Chiang Mai, Thailand. Of these, 70 were AFB-positive and 70 were AFB-negative samples. Conventional acid-fast bacilli (AFB) smear microscopy of all sputum samples was routinely performed by OPDC1 personnel using the Kinyoun staining method and reported according to standard AFB grading criteria. Specimens were processed using N-acetyl-L-cysteine–sodium hydroxide (NALC–NaOH). The processed pellets were cultured using either Lowenstein–Jensen (LJ) solid culture or a BACTEC MGIT 960 liquid culture system (Becton, Dickinson and Company, NJ, USA) and incubated at 37 °C for 8 weeks. Detection of the *Mycobacterium tuberculosis* complex (MTC) was confirmed using the STANDARD Q TB MPT64 antigen rapid test (SD BIOSENSOR, Suwon-si, Gyeonggi-do, Republic of Korea). Species-level identification of both the MTC and NTM was performed using the Molecular REBA Myco-ID assay (YD Diagnostics, Yongin, Gyeonggi-do, Republic of Korea), according to the manufacturer’s instructions.

### 2.3. DNA Extraction from Bacterial Strains and Sputum Samples

Genomic DNA from MTBC and NTM strains derived from the ODPC-1 was extracted using the Anyplex^TM^ MTB/NTM Real-Time Detection extraction reagents (Seegene, Seoul, Republic of Korea), in accordance with the manufacturer’s instructions. A loopful of mycobacterial culture was suspended in the provided DNA extraction solution, vortexed briefly, and then boiled at 100 °C for 20 min. After centrifugation at 13,000 rpm for 5 min, the supernatant-containing genomic DNA was transferred to a clean microcentrifuge tube and stored at −20 °C until further use. Genomic DNA from MTB isolates, provided by the DR-TB Fund, Siriraj Foundation, was extracted using the cetyltrimethylammonium bromide (CTAB) method and stored at −20 °C until further use.

For non-mycobacterial bacterial strains, DNA extraction was preceded by overnight bacterial culture on appropriate media under standard microbiological conditions. Non-fastidious bacteria were grown on Trypticase Soy agar (Oxoid Ltd., Basingstoke, Hampshire, UK) at 35–37 °C for 18–20 h in ambient air. Fastidious bacteria were cultured on 5% Sheep Blood agar or Chocolate agar plates (Biomedia [Thailand] Co., Ltd., Nonthaburi, Thailand) at 35–37 °C for 18–20 h in a 5–10% CO_2_ atmosphere.

DNA extraction from sputum specimens was also performed using the Anyplex MTB/NTM Real-Time Detection extraction reagents (Seegene, Seoul, Republic of Korea). Briefly, 100 µL of DNA extraction solution was added to the NALC-NaOH-treated sputum pellet and vortexed for 30 s. The mixture was then boiled at 100 °C for 20 min and centrifuged at 13,000 rpm for 5 min. The supernatant containing extracted DNA was collected and stored at −20 °C until further use.

### 2.4. Primer Design and Selection

To enable specific detection of MTB, four different RPA primer pairs (eight primers) were designed targeting the IS*6110* insertion element of *M. tuberculosis* H37Rv (GenBank accession no. NC_000962.3). The primer design was conducted in accordance with the assay design manual of the TwistAmp^®^ Basic Kit (TwistDx, Maidenhead, UK), utilizing the Primer-BLAST (NCBI, MD, USA)) to generate primers ranging from 25 to 27 base pairs (bp). The primer pairs were successfully designed to amplify the target regions, yielding expected amplicons of 200 to 300 bp. The specificity was assessed in silico using Primer-BLAST (NCBI) to confirm exclusive alignment of the primer sequences with MTBC genomes. The sequences were synthesized by Integrated DNA Technologies, Inc. (IDT, Newark, IA, USA).

Sixteen pairwise combinations of the eight newly designed primers were evaluated under standard RPA conditions as outlined in the assay design manual. The optimal primer pair was selected based on its amplification efficiency, clarity of product on agarose gel electrophoresis, and lack of nonspecific bands during preliminary RPA screening assays.

### 2.5. Optimization of the RPA Reaction

DNA amplification of the IS*6110* target was carried out using the RPA technique with the TwistAmp^®^ Basic Kit (TwistDx, Maidenhead, UK), in accordance with the manufacturer’s protocol. Each 25 μL reaction comprised 14.75 μL of rehydration buffer, 0.24–0.64 μM of both forward and reverse primers, 100 ng of DNA template, and 14–20 mM of magnesium acetate (MgOAc) (TwistDx, Maidenhead, UK). Sterile distilled water (SDW) was added to adjust the final volume to 25 μL. The reaction mixture was incubated under varying conditions—at 37–42 °C for durations ranging from 10 to 25 min—to determine the optimal amplification parameters. Following amplification, the RPA products were purified using the MAGTEC NeoClean DNA (Bioentist, Bangkok, Thailand), according to the manufacturer’s instructions. The purified products were then subjected to 2% agarose gel electrophoresis for visualization and subsequently quantified using the Syngene documentation system (Synoptics Ltd., Cambridge, UK).

### 2.6. Development and Optimization of a Colorimetric MTB Detection Assay Using RPA Combined with AuNPs

Synthesis of 15–20 nm sized citrate-capped AuNPs was conducted by the sodium citrate reduction of HAuCl_4_ with some modification [18]. Briefly, a boiling solution of HAuCl_4_ (1 mM, 100 mL) (Sigma-Aldrich, St. Louis, MO, USA) was prepared under vigorous stirring. Then, sodium citrate solution (38.8 mM, 10 mL) (RCI Labscan Ltd., Bangkok, Thailand) was rapidly added, and the mixture was boiled for another 15 min while stirring. The mixture then changed from pale yellow to a reddish wine-like color, suggesting the creation of nanoparticles. The wine-red solution was allowed to cool to room temperature (RT), stirred for another 10 min, and stored at 4 °C until use. The characterization of prepared AuNPs was previously performed [19].

To perform a visual detection method, the effect of NaCl concentrations (0–80 mM) on the aggregation of AuNPs was initially evaluated (Figure S1). This optimization was essential to determine the NaCl concentration at which AuNPs would aggregate in the absence of target DNA. Following amplification, the 25 μL RPA reaction was denatured at 95 °C for 10 min to release single-stranded DNA. Subsequently, 75 μL of AuNP solution was added and incubated at RT for 5 min. To induce AuNPs’ aggregation, 5 μL of 0.8 M NaCl was added, resulting in a final NaCl concentration of 40 mM. A persistent red color indicated successful target amplification and the presence of MTB DNA, whereas a color shift from red to purple denoted the absence of amplification, indicating a negative result for MTB. The outcomes of the reactions were observed by visual inspection and quantitative spectrophotometric analysis. Absorbance spectra were recorded over the range of 400–700 nm. Then, the ratio of A_530_/A_630_ was determined to quantify the degree of AuNP aggregation. A higher A_530_/A_630_ ratio corresponds to dispersed AuNPs (indicative of a positive result), while a lower ratio indicates aggregation (suggesting a negative result). The characteristics of AuNPs, including particle size, polydispersity index (PDI), and zeta potential (ZP) corresponding to the color change, were determined by dynamic light scattering (DLS) analysis. Each sample was diluted to a suitable concentration and measured using DLS with Zetasizer (Malvern Instruments, Worcestershire, UK). In addition, scanning transmission electron microscopy (STEM) analysis was performed to visualize the dispersion and aggregation behaviors of AuNPs following the colorimetric reaction. A drop of sample was stratified onto a carbon-coated copper grid and air-dried. The morphology of AuNPs was observed using the JSM-IT800 Ultrahigh Resolution Field Emission SEM (JEOL, Peabody, MA, USA).

### 2.7. Limit of Detection of the TB-GoldDx Assay

To establish the analytical sensitivity of the TB-GoldDx assay, genomic DNA from MTB H37Rv was serially diluted from an initial concentration of 100 ng/µL down to 0.0001 ng/µL using 10-fold dilutions. Each dilution was subjected to the TB-GoldDx protocol to identify the lowest concentration consistently producing a visible colorimetric change. The DNA concentrations were converted into an equivalent number of *Mycobacterium* bacilli based on the genome size of the MTB H37Rv strain [20].

### 2.8. Validation and Evaluation of the TB-GoldDx Assay

The TB-GoldDx assay was validated using a panel of 100 isolates, including 72 clinical MTB isolates, 13 NTM, and 15 non-mycobacterial isolates. Assay performance was assessed in comparison with conventional culture method combined with molecular identification (STANDARD Q TB MPT64 antigen rapid test and REBA Myco-ID assay) to assess diagnostic accuracy and concordance. Further evaluation was carried out on 140 blinded sputum specimens, including 70 AFB-positive and 70 AFB-negative samples. These specimens were tested with the TB-GoldDx assay, and the results were compared to those obtained from conventional AFB smear microscopy to assess diagnostic performance. Additionally, a subset of 83 sputum specimens was tested using a commercial LPA, the GenoType MTBDRplus VER 2.0 (MTBDRplusV2; Hain Lifescience GmbH, Nehren, Germany), enabling comparative evaluation of diagnostic concordance between TB-GoldDx and a standard molecular method.

Key diagnostic metrics, including the sensitivity, specificity, positive predictive value (PPV), negative predictive value (NPV), positive likelihood ratio (PLR), negative likelihood ratio (NLR), and diagnostic accuracy, were calculated along with their 95% confidence intervals (CIs) to assess statistical reliability using diagnostic test evaluation calculator version 23.2.8 [21]. In addition, comparisons of group means were conducted using a Wilcoxon Signed-Rank Test in SPSS Statistics version 30 (IBM Corp., Armonk, NY, USA). Statistical significance was defined as a *p*-value less than 0.05.

## 3. Results

### 3.1. Optimization of RPA Reaction

To establish specific RPA-based MTBC detection, primer selection was conducted using sixteen pairwise combinations of the eight newly designed primers. Among these, the combination of IS6110-F7 (5′GACCGAAGAATCCGCTGAGCTGAAG 3′) and IS6110-R7 (5′GTTGATGTGGTCGTAGTAGGTCGATG 3′) demonstrated the most robust amplification performance. Following extensive optimization, the optimal reaction conditions were established using 0.24 µM of each primer and 8 mM of MgOAc in the 25 µL reaction, with the incubation at 39 °C for 20 min. Using MTB H37Rv genomic DNA as a template, the assay consistently yielded a single band of RPA products of the expected size (261 bp), as confirmed by gel electrophoresis (Figure S2).

### 3.2. Development of TB-GoldDx for Detecting MTBC

The principle of the developed assay is based on the prevention of AuNP aggregation through the interaction with single-stranded DNA (ssDNA) generated by RPA. In positive samples containing MTBC DNA, the RPA amplifies the target sequence (IS*6110*) into double-stranded DNA (dsDNA), which is subsequently denatured at 95 °C for 10 min to yield ssDNA. The ssDNA adsorbs onto the surface of stabilized AuNPs, enhancing colloidal stability. This occurs because ssDNA can adsorb via van der Waals interactions between the nucleotide bases and the nanoparticles and shields them from salt-induced aggregation, whereas dsDNA cannot due to electrostatic repulsion between its phosphate backbone and the negatively charged AuNP surface [22]. Dispersed AuNPs (typically ~10–30 nm in diameter) exhibit surface plasmon resonance at ~ 520-530 nm, appearing red to the naked eye. In contrast, in negative samples, where ssDNA is absence, addition of NaCl reduces electrostatic repulsion, leading to AuNP aggregation and a visible color change from red to purple/blue, accompanied by a red shift of the SPR peak toward ~650 nm [23].

**Figure 1:**
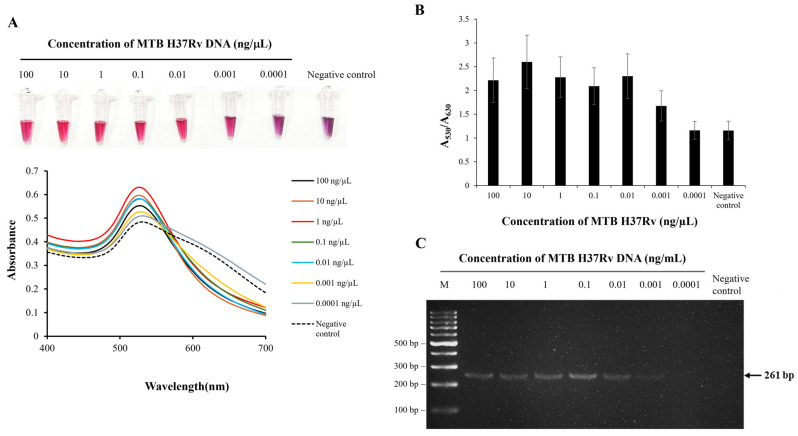
Detection limit of the TB-GoldDx assay for detecting *M. tuberculosis* H37Rv (MTB H37Rv) genomic DNA. Serial dilutions of genomic DNA were tested using the TB-GoldDx assay, with SDW served as the negative control. (**A**) Colorimetric detection of RPA products using unmodified AuNPs and the UV–Vis absorbance spectra of the AuNPs, observing changes in nanoparticle aggregation based on DNA input. (**B**) A quantitative A_530_/A_630_ absorbance ratio illustrating the degree of AuNP aggregation. (**C**) Agarose gel electrophoresis of RPA products confirming the amplification of the IS*6110* target (M: 100 bp DNA marker).

For AuNP-based detection, 25 μL of RPA product was denatured at 95 °C for 10 min to produce ssDNA, followed by 75 μL AuNP solution and a 5 min incubation at room temperature. Aggregation was triggered by the addition of 5 μL of 0.8 M NaCl. A red color indicated the successful amplification of IS*6110* in samples containing MTB genomic DNA, while a red-to-purple shift signified a negative result. Under these conditions, the TB-GoldDx assay demonstrated an LOD of 0.001 ng of *Mycobacterium tuberculosis* H37Rv DNA, equivalent to approximately 210 bacilli per 25 μL reaction. This result was validated through a shift in the UV–Vis absorbance spectrum of AuNPs from 530 nm to 630 nm, along with a quantitative A_530_/A_630_ absorbance ratio corresponding to the degree of AuNP aggregation (Figure 1A,B). Confirmation was further supported by 2% agarose gel electrophoresis of the RPA products (Figure 1C).

**Figure 2:**
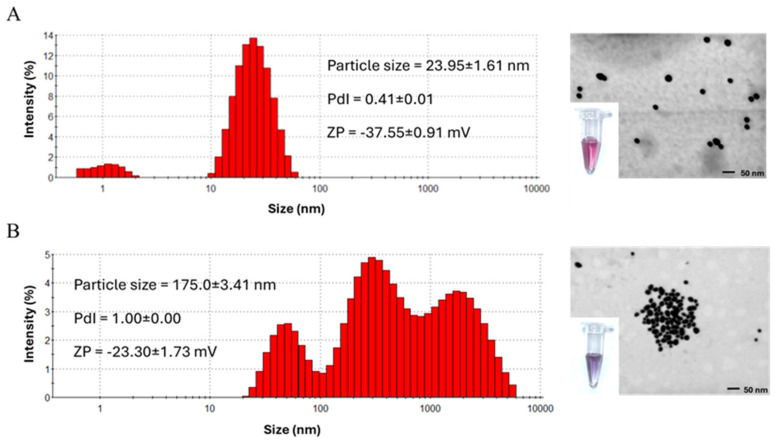
Characteristics of AuNPs corresponding to the observed color change. Dispersion and aggregation behaviors of AuNPs with (**A**) RPA products from *M. tuberculosis* H37Rv genomic DNA (red) and (**B**) non-target amplification products (purple) after NaCl addition, analyzed by dynamic light scattering (DLS) and scanning transmission electron microscopy (STEM).

To confirm the physicochemical properties of AuNPs corresponding to the observed color change from red to purple, they were characterized by dynamic light scattering (DLS) and scanning transmission electron microscopy (STEM). Colorimetric detection of RPA products from MTB H37Rv genomic DNA maintained red-colored colloidal gold after salt induction, suggesting preserved dispersion and a spherical shape, as shown by STEM images. Additionally, DLS measurements exhibited relatively uniform AuNPs with a size diameter of approximately 23.95 ± 1.61 nm, PDI of 0.41 ± 0.01, and surface charge of −37.55 ± 0.91 mV. Conversely, in the absence of target amplification, NaCl-triggered aggregation of AuNPs led to a color shift to purple. STEM imaging confirmed the formation of AuNP clustering. DLS analysis further revealed an increase in the hydrodynamic size (175.0 ± 3.41 nm), a high PDI (1.00), and a decreased ZP (−23.30 ± 1.73 mV), indicating loss of colloidal stability and the aggregation state (Figure 2).

### 3.3. Validation of TB-GoldDx Assay for Detecting Clinical MTB Isolates

**Figure 3:**
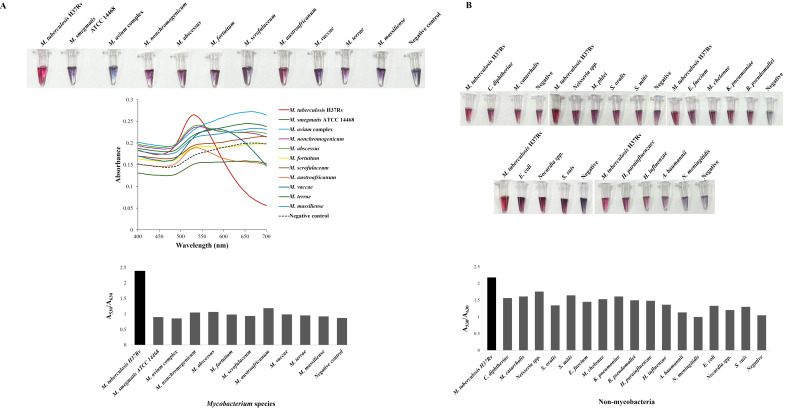
Validation of the TB-GoldDx assay using genomic DNA from various nontuberculous mycobacterial and non-mycobacterial species. DNA from *M. tuberculosis* H37Rv (MTB H37Rv) and SDW served as positive and negative controls, respectively. (**A**) Colorimetric detection of RPA products using unmodified AuNPs, with UV–Vis absorbance spectra reflecting nanoparticle aggregation based on RPA results with NTM DNA and a quantitative A_530_/A_630_ absorbance ratio for distinguishing MTB from NTM species. (**B**) Colorimetric detection of RPA products using unmodified AuNPs based on RPA results with non-mycobacterial DNA and a quantitative A_530_/A_630_ absorbance ratio for distinguishing MTB H37Rv from non-mycobacterial species.

The diagnostic performance of the TB-GoldDx assay was evaluated using 100 genomic DNA samples from 72 clinical MTB isolates and 28 nontuberculous bacterial species—including 13 NTM as well as 15 non-mycobacterial species associated with the respiratory tract—encompassing both commensals and pathogens. The assay exhibited no cross-reactivity with non-target mycobacterial or non-mycobacterial bacterial species, achieving specificity of 100% (Figure 3, Table 2).

**Figure 4:**
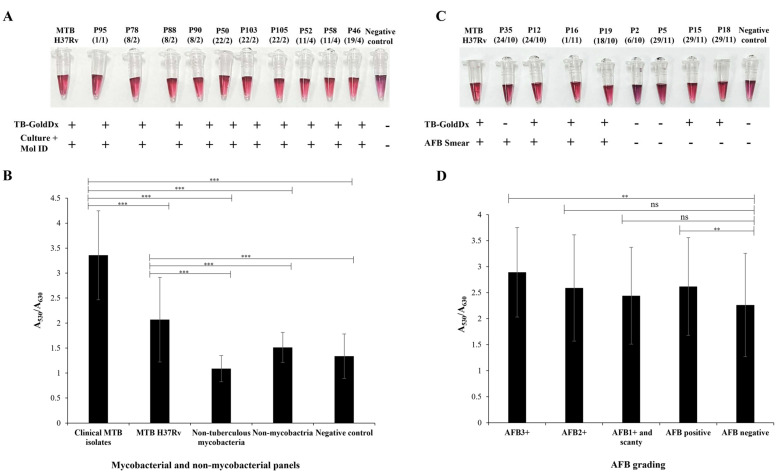
Visual interpretation of the TB-GoldDx assay and quantitative analysis of the A_530_/A_630_ absorbance ratio to evaluate the performance of the TB-GoldDx assay. Genomic DNA from *M. tuberculosis* H37Rv (MTB H37Rv) and SDW served as the positive and negative controls, respectively. (**A**) Representative interpretation of the TB-GoldDx assay for clinical MTB DNA samples compared with MTB identification using culture combined with molecular identification (Culture + Mol ID). (**B**) Differentiation among 100 DNA samples: clinical MTB strains (n = 72), nontuberculous mycobacteria (NTM; n = 13), and non-mycobacterial species (n = 15). (**C**) Representative interpretation of the TB-GoldDx assay for DNA samples directly extracted from sputum specimens compared with acid-fast bacilli smear microscopy (AFB smear). (**D**) Assessment of 140 DNA samples directly extracted from sputum specimens categorized by AFB grading levels: AFB-positive (n = 70) (comprising 22 AFB3+, 17 AFB2+, and 31 AFB1+/scanty) versus AFB-negative (n = 70). Statistical significance was indicated as follows: *p*-value < 0.01 (**), and *p*-value < 0.001 (***); “ns” indicates not significant.

Validation with clinical isolates revealed that the TB-GoldDx assay demonstrated a sensitivity of 95.83% [95%CI: 88.30–99.13%], correctly identifying 69 out of 72 MTBC samples, with three false negatives. It also exhibited a PPV of 100% [95%CI: 94.79–100.00%], and a negative predictive value (NPV) of 85.71% [95%CI: 75.51–96.58%] (Table 2). The prominent red color in positive samples was visually detected by TB-GoldDx as it was shown in represented DNA samples extracted from culture and molecular identification-confirmed MTB isolates (Figure 4A). Additionally, the A_530_/A_630_ absorbance ratio provided a quantitative differentiation between clinical MTB strains and the positive control (MTB H37Rv), as compared to NTM species and the negative control, with a *p*-value less than 0.001 (Figure 4B).

### 3.4. Evaluation of TB-GoldDx for Detecting MTBC from Sputum Specimens

The performance of the TB-GoldDx assay was evaluated using DNA extracted from 140 sputum specimens from suspected pulmonary TB cases in northern Thailand. MTBC detection was visually interpreted via AuNP colorimetric change: a persistent red color indicated a positive MTBC result, while a shift to purple denoted a negative outcome (Figure 4C). Quantitative analysis (Figure 4D) further demonstrated the distinct differences among clinical sputum samples stratified by AFB grading (3+, 2+, 1+, and scanty) compared to AFB-negative specimens, based on the A_530_/A_630_ absorbance ratio. Using the Wilcoxon Signed-Rank Test, statistically significant differences in the average A_530_/A_630_ absorbance ratios were observed when comparing AFB-positive samples and AFB 3+ samples to AFB-negative samples (*p*-values < 0.01).

Compared with the AFB smear results, the assay demonstrated an overall sensitivity of 81.43% [95% CI: 70.34–89.72%] and specificity of 58.57% [95% CI: 46.17–70.23%] (Table 2). Notably, assay performance improved in specimens graded AFB 3+, with a sensitivity of 95.45%. Reduced detection efficiency was observed in specimens graded AFB 2+ and 1+/scanty with the averaged sensitivity of 75.00% [95%CI: 60.40–86.36%]. Additionally, TB-GoldDx was evaluated by comparing its results with those of the MTBDRplusV2 LPA, using 83 sputum specimens. It showed a sensitivity of 82.98% [95% CI: 69.19–92.35%] and a specificity of 55.56% [95% CI: 38.10–72.06%] as shown in Table 2.

To benchmark the performance of TB-GoldDx against the WHO-recommended MTBDRplusV2 LPA, a comparative analysis was conducted on a subset of 83 sputum specimens, using AFB smear microscopy as the reference standard. Both TB-GoldDx and the LPA demonstrated 100% sensitivity in detecting MTB in AFB 3+ samples (n = 13). Overall, TB-GoldDx achieved a sensitivity of 84.78%, slightly higher than that of the LPA (82.61%). However, the TB-GoldDx assay exhibited a lower specificity of 54.05%, compared to 78.38% for the LPA. As a result, the overall performance, reflected by the diagnostic accuracy (71% vs. 80.72%), PLR (1,85 vs. 3.82), and NLR (0.28 vs. 0.22), suggests the moderate diagnostic effectiveness of the TB-GoldDx assay compared to the LPA (Table 3).

## 4. Discussion

This study demonstrates the potential of the AuNP-integrated RPA assay, termed TB-GoldDx, for rapid, sensitive, and specific detection of MTB. Designed to target the multiple copies’ insertion sequence, IS*6110*, the TB-GoldDx assay provides a visual readout based on colorimetric detection suitable for POC use in low-resource settings. The RPA-based assay was successfully developed using a newly designed primer set, highly specific to MTBC. The optimal conditions identified, including 39 °C, 0.24 µM primer concentration, 8 mM MgOAc, and 20 min incubation, highlight the assay’s simplicity and rapid turnaround time. Unlike PCR, real-time PCR, or real-time RPA-based assays reported, TB-GoldDx is operated through a simple isothermal RPA at a constant low temperature, eliminating the need for thermocyclers or complex post-amplification steps, thereby significantly reducing cost and procedural complexity.

Hussain et al. employed simple unmodified AuNPs for colorimetric detection of TB, with detection limits of 1 ng via PCR and 40 ng from crude genomic DNA. Although offering high concordance with BACTEC and nested PCR, the assay required thermal cycling. Additionally, the relatively high LOD, for both PCR-amplified DNA and crude samples, may limit its utility in detecting low-bacillary-load cases [24]. Similarly, Kooti et al. developed a colorimetric AuNP-based biosensor for TB detection in sputum, reporting 100% sensitivity and specificity across 52 clinical samples and an LOD of 4.8 × 10^−3^ ng per reaction. However, the method included a PCR step not accounted for in the reported 15 min readout. Its dependence on thermal cycling also restricts its suitability for POC applications [17].

Previous studies integrating RPA with AuNPs revealed varying trade-offs in sensitivity, ease of use, and readiness for use in field settings. A comparative summary of RPA-based MTB detection assays with the TB-GoldDx assay are shown in the Supplementary Table S1. For example, Singpanomchai et al. established an RPA-based assay targeting IS*6110* and IS*1081*, incorporating SYBR Green I for rapid and visual detection of MTB. The assay achieved detection limits of 0.5 ng for IS*6110* and 0.05 ng for IS*1081*, with reported sensitivities between ~90 and 95% and specificities approaching 100%, using genomic DNA from MTB strains [11]. Despite its strong analytical performance, the reliance on SYBR Green I fluorescence, though visible to the naked eye, may offer less visual stability and specificity compared to AuNP-based detection platforms. Due to unique physicochemical properties of AuNPs, TB-GoldDx provided a clear and reliable visual readout, demonstrated higher sensitivity (LOD: 0.001 ng), and achieved 100% specificity across 28 nontuberculous bacterial species. More recently, Zhang et al. developed a highly MTB-specific assay based on an asymmetric RPA-AuNP-CRISPR-Cas12a assay with an LOD as low as 1 copy/μL [25]. This study demonstrated that integrating CRISPR-Cas systems with RPA/AuNP assays can markedly enhance sensitivity. Its single-tube design and constant incubation at 40 °C for 40 min minimized the risk of aerosol contamination. The PAM-free CRISPR-Cas12a system integrated with asymmetry RPA enabled rapid and highly accurate detection of MTBC, and this sophisticated dual-mode readout platform is suitable for direct TB diagnosis in sputum specimens. The development of a POC assay similar to this platform, however, requires careful optimization of reaction conditions and additional reagents, and it may increase the assay cost, thereby reducing accessibility in resource-limited settings. By contrast, our assay was designed to provide a simple, cost-effective, and rapid point-of-care diagnostic tool while maintaining acceptable performance for clinical application.

In clinical validation, TB-GoldDx was evaluated across 140 sputum samples from suspected pulmonary TB patients in northern Thailand. The assay demonstrated an overall sensitivity of 81.43% relative to smear microscopy, with sensitivity rising to 95.45% (n = 140) to 100% (n = 83) in AFB 3+ samples, reflecting enhanced performance in high-bacillary-load conditions (Table 2 and Table 3). These results are comparable to the 82.61% sensitivity observed with the MTBDRplusV2 LPA, when tested on the same sample set (Table 3). Furthermore, the findings were consistent with the pooled sensitivity of three commercial LPAs, including Hain Genotype MTBDRplusV1, MTBDRplusV2, and Nipro NTM+MDRTB, estimated at approximately 94% for smear-positive TB [26]. Deng et al. similarly reported an LPA sensitivity of 87%, [95%CI: 84–90%] for pulmonary TB detection using sputum samples in a clinical study conducted in China. The study also demonstrated a pooled specificity of 94% (95% CI: 92–95%), underscoring the high diagnostic accuracy of the LPA [27]. However, our study revealed lower specificities: 78.38% for the LPA and only 54.05% for the TB-GoldDx assay. This discrepancy may be attributable to several factors, including geographic strain variation, variability in sputum composition, or cross-reactivity with nontuberculous bacterial species prevalent in the local setting.

LPAs achieve high specificity primarily through the amplification of target sequences, followed by hybridization with highly specific probes, which minimize cross reactivity. In contrast, TB-GoldDx employs an unmodified AuNP-based detection system, which offers simplicity and cost-efficiency but may be more prone to non-specific interactions. The use of thiol-modified probe-conjugated AuNPs has been shown to enhance the specificity of the AuNP-based assay for MTB detection [17]. However, this approach would increase both the cost and design complexity. The recent findings of Fukuzumi et al. further emphasize the critical role of surface chemistry and probe configuration in nanoparticle-based biosensing. Their study demonstrated that the density and conformational structure of DNA immobilized on AuNPs significantly influence detection sensitivity—lower probe density favored improved sensitivity for linear DNA while higher density benefitted more rigid stem-loop structures. These insights underscore the importance of rational probe design and surface optimization when developing ssDNA-AuNP biosensors [28]. Standard molecular diagnostic platforms such as the GenoType Mycobacterium CM, Mycobacterium AS, NTM-DR, and MTBDRplus (Hain LifeScience, Nehren, Germany) LPAs require multiple processing steps, including DNA extraction, amplification, hybridization, and result interpretation. These workflows typically require at least 5 h and are generally limited to centralized laboratories. By comparison, TB-GoldDx delivers comparable diagnostic performance in just less than 1 h, without the need for specialized equipment.

Although TB-GoldDx demonstrates adequate sensitivity for detecting MTBC in culture-based samples, its performance is notably reduced in direct detection from sputum specimens, particularly those with low bacillary loads. This limitation may restrict its applicability in certain tuberculosis cases, including early-stage infections, pediatric presentations, and immunocompromised populations [29,30,31]. Previous studies have shown that integrating the CRISPR-Cas (Clustered Regularly Interspaced Short Palindromic Repeats–CRISPR-associated) protein system with RPA technology significantly enhances the sensitivity of tuberculosis testing, particularly in samples with low target abundance. For instance, Zhang et al. demonstrated that integrating DNA-functionalized AuNPs with asymmetric RPA-triggered, PAM-free CRISPR yielded an LOD of 1 copy/μL, achieved 100% specificity concordant with qPCR and exhibited no cross-reactivity with non-TB pathogens using clinical sputum samples [25].

Recently, Compiro et al. introduced the MyTRACK assay, which combines CRISPR-Cas12a with RPA to detect and differentiate clinically relevant *Mycobacterium* species, including MTB, demonstrating an LOD of 1–100 copies per reaction and high sensitivity and specificity validated in clinical samples [32]. Furthermore, Xiao et al. demonstrated that incorporating graphene oxide (GO) improves the specificity of RPA-based amplification. Their study also employed a CRISPR–Cas12a-based combination with multiplex detection of IS*6110* and IS*1081* to enhance the sensitivity for MTBC detection. The resulting MCMD assay achieved a 4 copies/μL detection limit, showed no cross-reactivity with non-MTBC bacterial strains, and outperformed Xpert MTB/RIF with 74.8% sensitivity and 100% specificity in 107 clinical samples (vs. 63.6%, 100%) [33].

In addition, specimen preparation also plays a crucial role in molecular assay sensitivity. Reed et al. introduced the XtracTB assay that combines sequence-specific capture with qPCR targeting IS*6110* and *senX3-regX3* to minimize qPCR inhibitors and enhance sensitivity. The assay achieved an LOD of 5 MTB genomic copies/mL and demonstrated 88.6% sensitivity in smear-negative, culture-positive TB cases—markedly higher than the 67% reported for the Xpert^®^ MTB/RIF assay. Additionally, the assay demonstrated 94.9% sensitivity and 100% specificity in 140 sputum samples [34]. However, despite its strong diagnostic performance, the design and procedural complexities may not be well aligned with POC TB diagnostics.

Based on the preceding discussion, the proposed modifications could significantly enhance the diagnostic efficiency of the TB-GoldDx assay. However, it is crucial to maintain its current configuration, which effectively balances speed, simplicity, and user accessibility. By enabling rapid visual detection without the need for specialized instrumentation, TB-GoldDx represents a viable cost-effective alternative (approximately USD 8 per test) for confirming MTB detection in AFB-positive specimens. It may serve as either a replacement or complementary approach to established molecular diagnostic platforms, particularly in resource-constrained settings. Prospective enhancements to the TB-GoldDx platform may include the incorporation of thiol-modified probe-conjugated AuNPs and graphene oxide, along with artificial intelligence (AI)-assisted readout technologies to improve assay specificity and reduce subjectivity in result interpretation. Comprehensive comparative studies evaluating TB-GoldDx against established diagnostic standards, such as the Xpert MTB/RIF assay, across diverse clinical and operational contexts shall provide valuable insight into its real-world performance and potential for broader implementation. Ultimately, the continued refinement of TB-GoldDx toward enhanced sensitivity, diagnostic robustness, and operational accessibility may contribute meaningfully to narrowing diagnostic gaps in underserved populations and advancing the objectives of the World Health Organization’s End TB Strategy.

## 5. Conclusions

The TB-GoldDx assay, which integrates RPA with unmodified AuNP-based colorimetric detection, presents a rapid, sensitive, and equipment-free approach for MTB detection. While the assay exhibited high sensitivity and specificity with DNA from cultured MTB isolates, its performance was moderately reduced in sputum samples. Nonetheless, TB-GoldDx shows strong promise as a frontline screening tool for high-bacterial-load TB cases, particularly in resource-constrained settings where access to complex molecular diagnostics is limited. Its affordability, operational simplicity, and rapid turnaround time underscore its suitability for decentralized and field-based use. Future improvements are expected to further enhance its sensitivity and specificity while minimizing interpretation variability. These advancements will support its evolution into a robust POC diagnostic tool aligned with global TB elimination strategies.

## Figures and Tables

**Table 1:** Tuberculous and nontuberculous bacterial species used in this study.

Mycobacterial Species	Source (No.)	Non-Mycobacterial Species	Source (No.)
*Mycobacterium tuberculosis* complex (MTC)		Respiratory pathogens and normal flora	
*Mycobacterium tuberculosis* H37Rv (ATCC 27294)	CMB (1)	*Haemophilus influenzae*	CMB (1)
*Clinical M. tuberculosis* isolates (n = 72)	ODPC-1 (20), DR-TB (52)	*Moraxella catarrhalis*	CMB (1)
Nontuberculous mycobacteria (NTM)		*Enterococcus faecium*	CMB (1)
*Mycobacterium smegmatis* ATCC 14468	CMB (1)	*Acinetobacter baumannii*	CMB (1)
*Mycobacterium avium* complex	CMB (1)	*Burkholderia pseudomallei*	CMB (1)
*Mycobacterium chelonae*	CMB (1)	*Klebsiella pneumoniae*	CMB (1)
*Mycobacterium phlei*	CMB (1)	*Escherichia coli*	CMB (1)
*Mycobacterium avium-intracellulare*	CMB (1)	*Neisseria meningitidis*	CMB (1)
*Mycobacterium nonchromogenicum*	CMB (1)	*Corynebacterium diphtheriae*	CMB (1)
*Mycobacterium abscessus*	CMB (1)	Respiratory bacterial flora	
*Mycobacterium fortuitum*	CMB (1)	*Haemophilus parainfluenzae*	CMB (1)
*Mycobacterium scrofulaceum*	CMB (1)	alpha-*Streptococcus* sp.	CMB (1)
*Mycobacterium austroafricanum*	CMB (1)	*Streptococcus oralis*	CMB (1)
*Mycobacterium vaccae*	CMB (1)	*Streptococcus mitis*	CMB (1)
*Mycobacterium terrae*	CMB (1)	*Nocardia* sp.	CMB (1)
*Mycobacterium massiliense*	CMB (1)	*Neisseria* sp.	CMB (1)

**Table 2:** The efficacy of the TB-GoldDx assay in detecting *Mycobacterium tuberculosis* (MTB). Evaluation against the culture method combined with molecular species identification (Culture + Mol ID) on tuberculous and nontuberculous bacteria and the comparison of TB-GoldDx to AFB smear microscopy and the MTBDRplusV2 LPA on sputum specimens.

Assays		TB-GoldDx Assay	
		*Sensitivity (%)*	*Specificity (%)*
Culture + Mol ID 100 MTB isolates	MTB-positive (72)	95.83 (69/72) [95% CI: 88.30–99.13]	100 (28/28) [95% CI: 87.66–100.00]
	MTB-negative (28)		
Smear microscopy 140 sputum samples	AFB 3+ (22)	95.45 (21/22)	-
	AFB 2+ (17)	70.59 (12/17)	-
	AFB 1+/ Scanty (31)	77.42 (24/31)	-
	AFB-positive (70)	81.43 (57/70) [95% CI: 70.34–89.72]	58.57 (41/70) [95% CI: 46.17–70.23]
	AFB-negative (70)		
LPA 83 sputum samples	LPA-positive (47)	82.98 (39/47) [95% CI: 69.19–92.35]	55.56 (20/36) [95% CI: 38.10–72.06]
	LPA-negative (36)		

**Table 3:** Performance of TB-GoldDx assay compared with MTBDRplusV2 LPA using sputum specimens.

Smear Grade	Statistic	TB-GoldDx Assay	LPA
AFB 3+ (13)	*Sensitivity (%)*	100 (13/13)	100 (13/13)
AFB 2+ (6)	*Sensitivity (%)*	88.33 (5/6)	88.33 (5/6)
AFB 1+/ Scanty (27)	*Sensitivity (%)*	77.78 (21/27)	74.07 (20/27)
AFB-positive (46)	*Sensitivity (%)*	84.78 (39/46) [95% CI: 71.78–92.43]	82.61 (38/46) [95%CI: 69.28–90.91]
AFB-negative (37)	*Specificity (%)*	54.05 (20/37) [95% CI: 38.38–68.96]	78.38 (29/37) [95% CI: 62.80–88.61]
*Positive Likelihood Ratio (PLR)*		1.85 [95% CI: 1.27–2.67]	3.82 [95% CI: 2.04–7.16]
*Negative Likelihood Ratio (NLR)*		0.28 [95% CI: 0.13–0.59]	0.22 [95% CI: 0.12–0.43]
*Overall Accuracy (%)*		71.08 [95% CI: 60.09–80.52]	80.72 [95% CI: 70.59–88.56]

## Data Availability

The data supporting the findings of this study include laboratory results from tuberculosis patients and are not publicly available due to privacy and ethical restrictions.

## Supplementary Materials

The following supporting information can be downloaded at https://www.mdpi.com/article/10.3390/bios15090607/s1, Figure S1: Determination of NaCl concentration. (A) Colorimetric detection of RPA products using unmodified AuNPs; (B) The UV-Vis absorbance spectra of the AuNP aggregation; and (C) A quantitative A_530_/A_630_ absorbance ratio illustrating the degree of AuNP aggregation; Figure S2: Optimization of the RPA assay demonstrated by agarose gel electrophoresis of RPA products. M: 100-bp DNA marker; MTB: *M. tuberculosis* H37Rv genomic DNA; and N: negative control. (A) Representative primers selection experiment performed using forward primers F6 and F7 and reverse primers R3, R4, R6, and R7; (B) Optimization of reaction temperatures; (C) Optimization of primer concentration; (D) Optimization of MgAOc concentration; (E) Optimization of incubation time; and (F) RPA products under optimal conditions. Red boxes indicate achieved optimal condition; Table S1. A comparative summary of RPA-based MTB detection assays with TB-GoldDx assay.

## Abbreviations

The following abbreviations are used in this manuscript:

LPA	Line probe assay
PCR	Polymerase chain reaction
Mol ID	Molecular mycobacterial species identification
MTB	*Mycobacterium tuberculosis*
MTC	*Mycobacterium tuberculosis* complex
