# Supplementary material for: Gold Nanoparticle-Enhanced Recombinase Polymerase Amplification for Rapid Visual Detection of Mycobacterium tuberculosis
Source: Biosensors (Basel). 2025 Sep 15;15(9):607. doi: 10.3390/bios15090607 (PMC12467982; doi:10.3390/bios15090607)
Supplement: Supplementary file 1 [file biosensors-15-00607-s001.zip › biosensors-3820254-supplementary.pdf]

Supplementary Materials

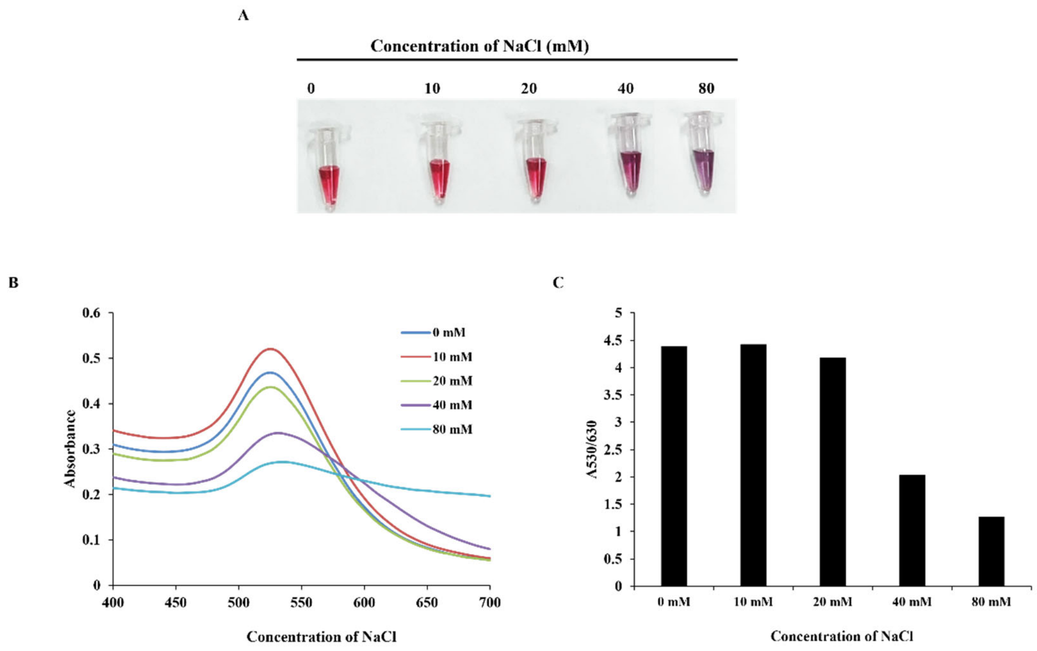

Figure S1. Determination of NaCl concentration. (A) Colorimetric detection of RPA products using unmodified AuNPs; (B) The UV-Vis absorbance spectra of the AuNP aggregation; and (C) A quantitative A_{530}/A_{630} absorbance ratio illustrating the degree of AuNP aggregation

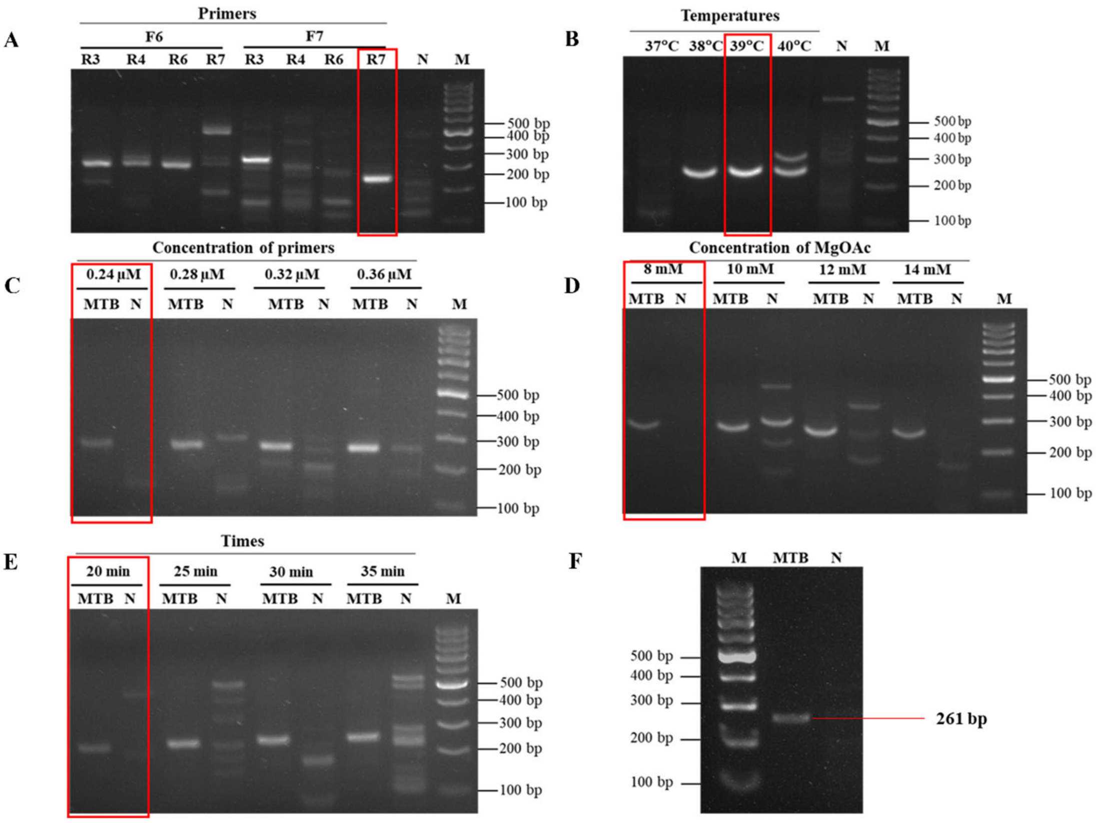

Figure S2. Optimization of the RPA assay demonstrated by agarose gel electrophoresis of RPA products. M: 100-bp DNA marker; MTB: *M. tuberculosis* H37Rv genomic DNA; and N: negative control. (A) Representative primers selection experiment performed using forward primers F6 and F7 and reverse primers R3, R4, R6, and R7; (B) Optimization of reaction temperatures; (C) Optimization of primer concentration; (D) Optimization of MgAOc concentration; (E) Optimization of incubation time; and (F) RPA products under optimal conditions. Red boxes indicate achieved optimal condition.

Table S1. A comparative summary of RPA-based MTB detection assays with TB-GoldDx assay.

Assay/ Method	Principle	Detection system/ Time	Target	LOD	Samples	Sensitivity	Specificity	Reference
TB-GoldDx	RPA + unmodified AuNPs	Colorimetry<br>~ 40 min	IS*6110*	0.001 ng (~210 bacilli) per 25-µL reaction	Culture (n =100)<br>(72 Mtb and 13 NTM 15 non-mycobacteria)<br><br>Sputum (n =140)	95.83% vs. Culture+ Mol ID<br><br>81.43% vs AFB smear<br>82.98% vs. LPA	100% vs. Culture+ Mol ID<br><br>58.57% vs. AFB smear<br>55.56% vs. LPA	This study
RPA-S	RPA + SYBR Green I	Fluorescence<br>15 min RPA	IS*6110* or IS*1081*	0.05 ng<br>0.5 ng	Culture (n = 146)<br>(146 Mtb and 24 NTM isolates)	97.95% or 99.32%	100% vs.	Singpanomchai et al. (2019) [11]
RT RPA and RPA-LF	0 Real time RPA<br>0 RPA-Lateral flow	Fluorescence and Lateral flow<br>~ 40 min	IS*1081* or IS*6110*	25 fg/µL<br>5 fg/µL	Sputum (n =62)	85.1%<br><br>53.2%	93.3%<br><br>93.3%	Sciaudone et al. (2023) [11]
MCMD	0 RPA-CRISPR–Cas12a-based + Real time fluorescence<br>0 Lateral flow	Fluorescence and Lateral flow within 1h	IS*6110* and IS*1081*	4 copies/µL	Sputum<br>(n =147)	74.8%	100%	Xiao et al. (2023) [29]
Dual-mode MTB sensing	Asymmetric RPA-triggered PAM-free CRISPR system + DNA-functionalized AuNPs	Fluorescence and Colorimetry<br>30 min RPA + 40 min detection	N/A*	1 copy/µL	Sputum<br>(n =32)	100%	100%	Zhang et al. (2025) [22]
MyTRACK	RPA-CRISPR system	Fluorescence<br>30 min RPA + ~ 20 min detection	*rpoB*	1–100 copies/ reaction	Culture (n = 82)<br>Sputum (n = 20)	92.59-100%<br>-	100%<br>-	Compiro et al. (2025) [28]

*N/A: The assay target is described in the supplementary materials; however, access to the document is currently restricted.
